# Unique Genomic Landscape of High-Grade Neuroendocrine Cervical Carcinoma: Implications for Rethinking Current Treatment Paradigms

**DOI:** 10.1200/PO.19.00248

**Published:** 2020-09-03

**Authors:** Ramez N. Eskander, Julia Elvin, Laurie Gay, Jeffrey S. Ross, Vincent A. Miller, Razelle Kurzrock

**Affiliations:** ^1^ Department of Obstetrics, Gynecology and Reproductive Sciences, Division of Gynecologic Oncology, University of California San Diego, La Jolla, CA; ^2^ Center for Personalized Cancer Therapy, University of California San Diego, La Jolla, CA; ^3^ Foundation Medicine, Cambridge, MA; ^4^ Upstate Medical University, Syracuse, NY; ^5^ Department of Medicine, Division of Hematology/Oncology, University of California San Diego, La Jolla, CA

## Abstract

**PURPOSE:**

High-grade neuroendocrine cervical cancer (HGNECC) is an uncommon malignancy with limited therapeutic options; treatment is patterned after the histologically similar small-cell lung cancer (SCLC). To better understand HGNECC biology, we report its genomic landscape.

**PATIENTS AND METHODS:**

Ninety-seven patients with HGNECC underwent comprehensive genomic profiling (182-315 genes). These results were subsequently compared with a cohort of 1,800 SCLCs.

**RESULTS:**

The median age of patients with HGNECC was 40.5 years; 83 patients (85.6%) harbored high-risk human papillomavirus (HPV). Overall, 294 genomic alterations (GAs) were identified (median, 2 GAs/sample; average, 3.0 GAs/sample, range, 0-25 GAs/sample) in 109 distinct genes. The most frequently altered genes were *PIK3CA* (19.6% of cohort), *MYC* (15.5%), *TP53* (15.5%), and *PTEN* (14.4%). *RB1* GAs occurred in 4% versus 32% of HPV-positive versus HPV-negative tumors (*P* < .0001). GAs in HGNECC involved the following pathways: PI3K/AKT/mTOR (41.2%); RAS/MEK (11.3%); homologous recombination (9.3%); and ERBB (7.2%). Two tumors (2.1%) had high tumor mutational burden (TMB; both with *MSH2* alterations); 16 (16.5%) had intermediate TMB. Seventy-one patients (73%) had ≥ 1 alteration that was theoretically druggable. Comparing HGNECC with SCLC, significant differences in TMB, microsatellite instability, HPV-positive status, and in *PIK3CA*, *MYC*, *PTEN, TP53*, *ARID1A*, and *RB1* alteration rates were found.

**CONCLUSION:**

This large cohort of patients with HGNECC demonstrated a genomic landscape distinct from SCLC, calling into question the biologic and therapeutic relevance of the histologic similarities between the entities. Furthermore, 73% of HGNECC tumors had potentially actionable alterations, suggesting novel treatment strategies for this aggressive malignancy.

## INTRODUCTION

The treatment of solid malignancies has evolved and is perhaps best exemplified by the approach to non–small-cell lung cancer, for which molecular characterization and use of targeted agents have emerged as standard therapeutic paradigms. Recently, The Cancer Genome Atlas (TCGA) completed and published the integrated genomic and molecular characterization of cervical cancer.^1^ In addition to data previously released for both ovarian (high-grade serous) and endometrial (endometrioid and serous) cancers, this publication completed the molecular and genomic evaluation of the most common gynecologic malignancies.^2,3^

**Context**

**Key Objective**
To define the molecular landscape of high-grade neuroendocrine cervical cancer in a large cohort of patients.
**Knowledge Generated**
High-grade neuroendocrine cervical cancer appears molecularly distinct from the histologically similar small-cell lung cancer. Up to 73% of patients’ samples harbored potentially actionable alterations, informing novel treatment strategies.
**Relevance**
Continued understanding of the molecular underpinnings of high-grade neuroendocrine cervical carcinoma will be critical to driving drug discovery for this disease.

Traditionally, cervical cancer clinical trials have excluded less common histologies such as high-grade neuroendocrine cervical carcinoma (HGNECC). Despite the low incidence of HGNECC (< 2% of all cervical cancers) the oncologic impact is significant because these tumors exhibit more aggressive clinical characteristics.^4,5^ Unfortunately, the 5-year overall survival rate for patients with early-stage disease is only a 36%, and those with metastatic spread face an even more dismal prognosis. Given these poor outcomes, patients with HGNECC represent an area of unmet clinical need.

Developing therapeutic options for patients with rare tumors is challenging, relying on international collaboration, as well as small case series or retrospective reports rather than prospective clinical trials. The current therapeutic paradigm for the treatment of HGNECC was adopted from the more common, morphologically similar, small-cell lung cancer (SCLC) and includes surgical resection if feasible, followed by platinum plus etoposide-based combination chemotherapy, and possibly radiation.^6,7^ There are few studies informing treatment of recurrent disease, and there are no drugs approved by the Food and Drug Administration (FDA) specifically for HGNECC.^8^

Recently, 2 reports of exceptional responses to immune checkpoint inhibition in patients with recurrent HGNECC were published.^9,10^ To better understand these responses and to identify molecular aberrations underlying this uncommon malignancy, we examined the genomic landscape of HGNECC. Here, we report the identified molecular alterations in a cohort of HGNECC specimens, several of which may serve as actionable therapeutic targets, and compare the genomic landscape of this disease to the histologically similar SCLC, which has been historically used to model treatment of HGNECC.

## PATIENTS AND METHODS

We evaluated a fully informative genomic profile of patients diagnosed with poorly differentiated (G3) neuroendocrine cervical carcinomas inclusive of both small- or large-cell subtypes (HGNECCs) whose cancers were submitted for hybrid capture–based next-generation sequencing (NGS) testing from March 2013 to December 2017 (N = 97). A cohort of 1,800 similarly tested cases of SCLC from the same period were subsequently evaluated to allow for comparison of genomic alterations (GAs). The submitting physicians provided specification of a poorly differentiated, neuroendocrine tumor type of cervical origin, which was then independently reviewed by a gynecologic pathologist (J.E.) to confirm high-grade neuroendocrine pathologic features in the pathology report and/or the representative sample of tumor submitted for sequencing (grade 3 cytomorphologic features, some component of small-cell or large-cell carcinoma histology, and/or positivity for neuroendocrine markers). The database was de-identified with only the diagnosis available. NGS data were generated by FoundationOne (Foundation Medicine; Cambridge, MA). The study was performed in accordance with University of California, San Diego, Institutional Review Board guidelines for a de-identified database. Approval for this study, including a waiver of informed consent and a Health Insurance Portability and Accountability Act waiver of authorization, were also obtained from the Western Institutional Review Board (Protocol No. 20152817).

### Tissue Samples and Mutational Analysis

Available tissue from diagnostic or therapeutic procedures was used to determine oncogenic molecular alterations. Sequencing information was collected on 97 patients with HGNECC and 1,800 with SCLC, whose formalin-fixed, paraffin-embedded tumor samples were submitted to Foundation Medicine for genomic profiling. The test sequences the entire coding region of 182 or, more recently, 236 or 315 cancer-related genes plus up to 47 introns of up to 19 genes often rearranged or altered in cancer to an average depth of coverage of > 500×.^11^ The pathologic diagnosis of each case was confirmed on routine hematoxylin- and eosin-stained slides and all samples forwarded for DNA extraction contained a minimum of 20% tumor nuclear area. Microsatellite instability (MSI) status was evaluable in 75 HGNECC and 1,573 SCLC cases.

The sequencing methods used for comprehensive genomic profiling have been validated and reported previously (Appendix).^12,13^ The optimized loci used to evaluate MSI status were selected from a total set of 1,897 that have adequate coverage on all versions of the assay. Each locus is intronic and has a reference repeat length of 10-20 bp, which allows for analysis with the read length used by FoundationOne testing. Principal components analysis is used to produce an NGS-based MSI score. There was no need to extend beyond the first principal component, because it explained approximately 50% of the total data variance, whereas none of the other principal components explained > 4% each. Ranges of the MSI score were assigned MSI-High (MSI-H), MSI ambiguous, or microsatellite stable (MSS). MSI-Low calls are not made because there was no gold-standard test set, but we presume such samples would significantly overlap with the MSI-ambiguous category reported here. For samples in which MSI-specific quality control criteria were not met (n = 22 HGNECC; n = 227 SCLC), a status of MSI unknown was assigned,^14^ and these cases were excluded from additional MSI analysis.

### Tumor Mutational Burden

The number of somatic mutations detected on NGS (interrogating up to 1.2 Mb of the genome) were quantified and that value extrapolated to the whole exome, using a validated algorithm.^15^ Alterations likely or known to be germline polymorphisms or bona fide oncogenic drivers were excluded. Tumor mutational burden (TMB) was measured in mutations per megabase. TMB levels were grouped into 3 bins: TMB-low (TMB-L; 1-5 mutations/Mb), intermediate (TMB-I; 6-19 mutations/Mb), and high (TMB-H; ≥ 20 mutations/Mb). The cutoff of 20 coding mutations/Mb is approximately equal to 400 nonsynonymous mutations per exome.^15^

### Human Papillomavirus Detection

In addition, the presence of high-risk human papillomavirus (HPV) was examined in submitted specimens, as previously reported.^16^ Hybrid-capture reagents included baits designed to capture unique regions of select viral genomes including HPV-16 and -18. Sequence read pairs were aligned to the reference genome of the respective viral genomes, and the number of pairs mapping to each viral genome was counted. A total HPV-16/18 aligned read count of ≥ 5 reads per million was considered a positive HPV status, and < 5 reads per million was considered HPV not detected.^16^

### End Points and Statistical Methods

Descriptive statistics were used to summarize the baseline patient characteristics. Fisher exact test was used to determine the association between categorical variables in univariate analysis and the Z-test was used to assess population differences, where appropriate. All tests were 2 sided. All statistical tests were carried out using GraphPad Prism, version 6.0 (GraphPad Software, San Diego, CA).

## RESULTS

### Characterization of GAs in HGNECC

**FIG 1.**
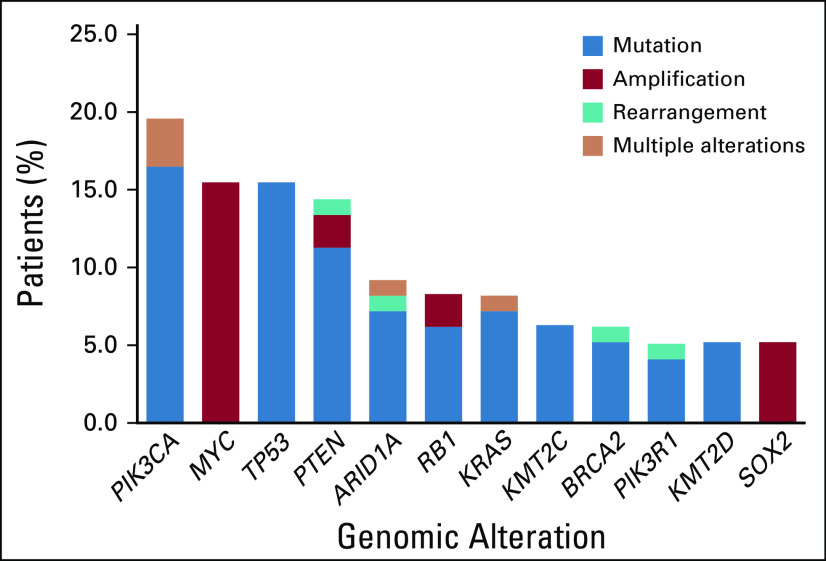
Most common genomic alterations in patients with high-grade neuroendocrine cervical cancer (HGNECC), reflected as percentage of patients (N = 97). Only genes altered in > 5% of samples are depicted. Variants of unknown significance excluded.

The median age of the cohort was 40.5 years (range, 25-77 years). Of the 97 patients, 83 were high-risk HPV positive (85.6%) and 14 were negative (14.4%). All samples were reflective of HGNECC, including both small-cell and large-cell HGNECC cases. Among the HGNECC cohort (N = 97), the most frequently identified GAs (discerned in > 10% of the cohort) involved *PIK3CA* (19.6% of patients), *MYC* (15.5%), *TP53* (15.5%), and *PTEN* (14.4%) (Fig 1; a detailed list of all GAs can be found in Appendix Table A1).

**FIG 2.**
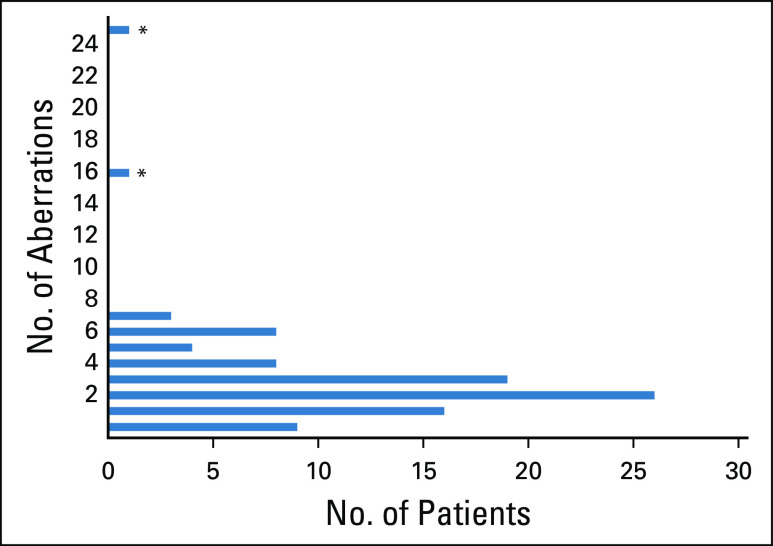
Number of reported genomic alterations per patient. Variants of unknown significance are excluded. (*) Tumor mutational burden high (*MSH2* mutation).

A total of 109 different genes were mutated in the 97 patient samples evaluated (variants of unknown significance were excluded from all analyses). The most frequently reported number of GAs per sample was 2, with a range of 0-25 (average, 3.0 GAs/sample; Fig 2). When evaluating TMB, 2 cases (2.1%) were TMB-H and 16 cases (16.5%) were TMB-I. Most patients’ tumors (n = 79; 81.4%) were TMB-L (Table 1). Nine patient samples had no known or likely GAs on comprehensive genomic profiling. Of the 88 patients who had an alteration, 72 had at least 1 alteration for which there currently existed an agent potentially targeting that alteration.

**TABLE 1.**
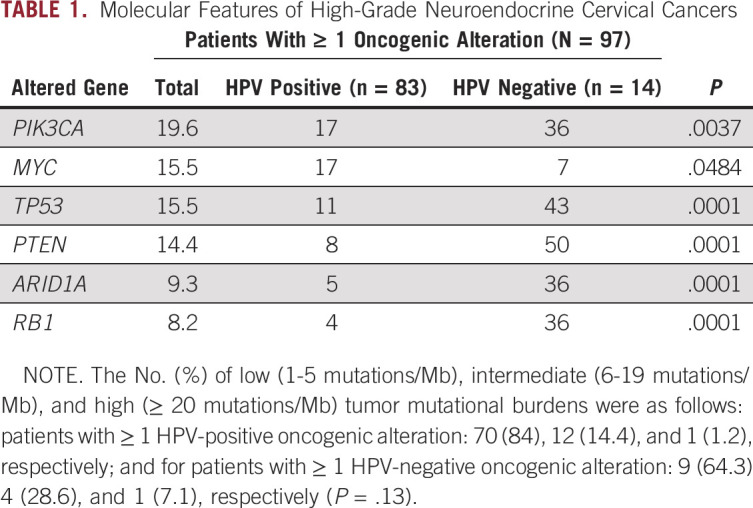
Molecular Features of High-Grade Neuroendocrine Cervical Cancers

### Genomics in HPV-Positive Versus -Negative Patients

When examining distribution of GAs on the basis of HPV detection status, a significant difference was identified in the frequency of several GAs, including *PIK3CA*, *TP53*, *PTEN*, *ARID1A*, and *RB1*, all of which were more frequent in the HPV-negative subgroup (Table 1). There was no difference in the frequency of TMB-H between HPV-positive and HPV-negative or unknown samples (*P* = .2691).

### Mismatch Repair Gene Aberrations Associated With MSI-High

Three cases (3%) had pathogenic *MSH2* alterations. Two of the 3 cases were both MSI-H and TMB-H and harbored the highest numbers of identifiable GAs in the cohort.^17,18^ The third case, with a nonsense mutation near the 3′ end of the coding sequence (MSH2 R929*), was MSS and TMB-L. The 2 MSH-2–mutant MSI-H cases were both of small-cell histology and accounted for all MSI-H cases out of the 75 cases of HGNECC with evaluable microsatellite status (2.7%).

### Less Frequent GAs

Additional genomic characterization was performed in which we specifically explored the homologous recombination deficiency (HRD), RAS, PI3K/AKT/mTOR, and ERBB pathways. Nine cases (9.3%) were had GAs in HRD-related genes, with the most frequent alterations noted in *BRCA2* (n = 6 of 9; 66.7%).^17,19^ Three additional patient samples had *BRCA1*, *ATM*, and *PALB2* mutations (n = 1 in each case) case (n = 3 of 9; 33.3%).

Eleven patient samples (11.3%) had alterations in the RAS pathway, with *KRAS* and *BRAF* mutations being the most frequent (72.7% [n = 8 of 11] and 27.3% [n = 3 of 11], respectively). Of the identified BRAF mutations, only 1 was a V600E alteration. Furthermore, a total of 40 patient samples (41.2%) harbored mutations in the PI3K/AKT/mTOR pathway; mutations in *PIK3CA* were identified in 47.5% of these samples (n = 19), and *PTEN* mutations were reported in 35% (n = 14). Last, 7 patients (7.2%) had mutations in the ERBB pathway, with *ERBB2* mutations occurring in tumors of 4 individuals (57.1%).

### Comparison of HGNECC and SCLC

Given the histologic similarity between SCLC and HGNECC, tumor samples from a cohort of 1,800 patients with SCLC were compared with the HGNECC samples (Table 2). The SCLC samples featured significantly lower frequencies of GAs in *PIK3CA*, *MYC*, and *ARID1A*. In contrast, the HGNECC samples featured significantly lower frequencies of GAs in *TP53* and *RB1*. High-risk HPV was identified in much less than 1% of SCLC tumor samples compared with 85.6% of HGNECC tumor samples. There was a single MSI-H SCLC case (n = 1 of 1,449; < 0.001%), whereas MSI-H status was found in 2 HGNECC cases (2.7%). Last, TMB was significantly higher in the SCLC samples compared with the HGNECC samples with respect to both intermediate and high TMB levels. The small-cell subset of HGNECC samples showed analogous gene mutation differences from SCLC.

**TABLE 2.**
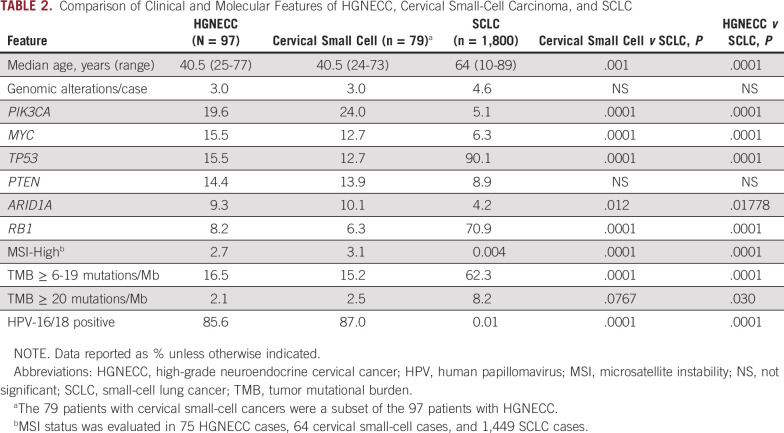
Comparison of Clinical and Molecular Features of HGNECC, Cervical Small-Cell Carcinoma, and SCLC

## DISCUSSION

Neuroendocrine carcinoma is an uncommon but aggressive variant accounting for approximately 1.5% of all newly diagnosed cervical cancers.^20^ The great majority of these lesions are high-grade large- or small-cell subtypes, with only rare reports of well-differentiated cervical carcinoid tumors.^20^ The treatment of patients with HGNECC remains clinically challenging, with limited response rates to chemotherapy; however, anecdotal reports of exceptional responders have been described.^9,10^

The paradigm for management of HGNECC has been informed by the treatment of the more commonly diagnosed (and histologically similar) SCLC, which accounts for approximately 15% of all lung cancer cases. In prior studies, whole-genome sequencing of 110 SCLC specimens identified essentially ubiquitous *TP53* and *RB1* inactivating mutations, with biallelic losses of each gene respectively in 100% and 93% of cases without chromothripsis.^21^

In an effort to better define the molecular landscape of HGNECC, we evaluated the comprehensive genomic profiling of 97 patient samples. The most frequently identified GA was *PIK3CA* mutation, occurring in 19.6% of submitted samples (n = 19). At least 1 characterized alteration was identified in 88 patient samples (90.7%) and of these, 72 had a potentially pharmacologically tractable alteration.

Interestingly, the frequency and distribution of GAs identified in this cohort of patients are similar and distinct from mutational patterns described in the more common HPV-related cervical cancer histologies.^1^ As detailed in TCGA’s integrated genomic characterization of cervical cancer (ie, squamous, adenocarcinoma, and adenosquamous histologies), mutations in the *PIK3CA* gene were the most frequently identified aberration, occurring in 26% of samples, approximating the nearly 20% rate in our cohort. In addition, significantly mutated genes reported by the TCGA, identified in similar proportions in this patient cohort, included *ARID1A* (7% in TCGA and 9.3% in our cohort) and *KRAS* (6% in TCGA and 8.2% in our cohort). Conversely, the examined neuroendocrine cohort had a greater frequency of *PTEN* mutations (8% in TCGA *v* 14.4% in our cohort). These molecular differences may be reflective of the varying histologies or, potentially, the differential high-risk HPV detection rates (85.6% in our cohort *v* 95% in the TCGA).^1^ Importantly, the high-risk HPV rate in our cohort should be interpreted with caution because the assay used has not undergone formal concordance study with gold standard tests such as hybrid capture and can detect only HPV 16/18.

Our own, much larger cohort of SCLC samples (n = 1,800) recapitulates prior studies and had a strikingly different molecular portfolio when compared with HGNECC samples. The frequency of *TP53* and *RB1* alterations in the SCLC cohort significantly exceeded that seen in our HGNECC cohort (15.5% and 8.2%, respectively), the HPV16/18 positive subset (11% and 4%, respectively), and the subset where HPV16/18 was not detected (43% and 36%, respectively; Table 2). Furthermore, mutations affecting the *NOTCH* pathway were identified in 25% of the examined SCLC samples; the *NOTCH* pathway is hypothesized to function as a regulator of neuroendocrine differentiation. In our examined HGNECC cohort, only 7 patients (7.2%) had *NOTCH* alterations. Alterations in *PIK3CA*, *MYC*, and *PTEN* were significantly more common in HGNECC when compared with SCLC (Table 2). MSI-H status was also more common in the HGNECC cohort whereas TMB-I/TMB-H was more common in SCLC (despite the lack of MSI-H status). Finally, HPV positivity was discerned in 85.6% of our HGNECC samples, but in only 0.01% of our SCLC samples (*P* < .0001). No parallels in molecular alterations were identified when comparing our findings for HGNECC with those of prior SCLC studies, supporting our premise that the similarity between these entities is largely morphologic and the treatment approaches for HGNECC can likely be improved through improved molecular granularity.

Despite the infrequency of HGNECC, the identification of potentially actionable GAs may inform treatment of a subset of patients with historically limited therapeutic options.^18,22-25^ In this cohort of patients, aberrations in the PI3K/AKT/mTOR pathway were commonly seen (*PIK3CA* [19.6%]; *PTEN* [14.4%]). The use of everolimus, or an alternate mTOR or PIK3CA inhibitor, may be considered in such circumstances, although the utility of a PIK3CA mutation in predicting response to single-agent everolimus in the presence of multiple GAs remains limited.^26,27^

Although less frequently identified, alterations in the HRD pathway were detected in 9.3% of patient samples, potentially supporting use of a poly-ADP ribose polymerase inhibitor. The identification of both TMB-H (n = 2) and GAs in mismatch repair genes (n = 3) may also inform the use of immune checkpoint inhibition.^28^ In May 2017, the FDA approved pembrolizumab for the treatment of mismatch repair–deficient or MSI-H solid tumors that progressed after prior therapy. This disease site–agnostic approval allows for a promising therapeutic option for patients with a previously unmet clinical need. More recently, the FDA accepted and granted priority review to a supplemental Biologics License Application for pembrolizumab for the treatment of adult and pediatric patients with unresectable or metastatic solid tumors with tissue TMB-H whose disease has progressed after prior treatment and who have no satisfactory alternative treatment options, supported by data from the phase II Keynote-158 trial. Notably, there are 2 published case reports of patients with recurrent, treatment-refractory HGNECC with exceptional and durable responses to checkpoint inhibition; 1 of these tumors was from our current HGNECC cohort and had a mismatch repair defect and the other lacked correlative genomic testing.^9,10^

Last, the identification of *ARID1A* (9.3%) and *SMARCA4* (4.1%) mutations may predict sensitivity to an alternate therapeutic strategy.^29^ Homeostasis requires balanced *ARID1A* and *EZH2* activity, facilitated via chromatin-mediated gene expression. Loss of *ARID1A* expression results in imbalanced EZH2 activity, and use of an EZH2 inhibitor such as tazemetostat may capitalize on this oncogene addiction. Importantly, 2 of the 4 SMARCA4 aberrations were identified in patients with MSI-H lesions, possibly reflecting that the SMARCA4 may be a passenger mutation resulting from the underlying MSI. Furthermore, of the 4 cases with SMARCA4 alterations, 1 was HPV-18 positive and another was p16 positive by immunohistochemical assessment.

Despite the large sample size and robust genomic data, this study has limitations. The retrospective design and use of archival tumor tissues from various time points during therapy may make interpretation of GAs difficult. In addition, the lack of demographic and clinical data, as well as treatment history, precludes exploratory assessments of response to a selected targeted agent. Last, HPV status was determined using molecular surrogates that differ from the assays used in clinical practice. It remains unclear if HPV infection is a prerequisite for neuroendocrine cervical carcinoma, although recent publications suggest > 85% of neuroendocrine cervical carcinomas are HPV positive, with HPV-16 and HPV-18 accounting for > 95% of the identified high-risk HPV strains.^30^

This report highlights the potential therapeutic utility of genomic testing in patients with this uncommon disease.^27^ Of interest, despite the histologic similarity between HGNECC and SCLC, which has led to the latter being used as a model for treating the former, the molecular portfolio of these 2 entities is strikingly different. Therefore, it is plausible that patients with HGNECC may benefit from alternative therapeutic strategies.

It is not anticipated that traditional prospective trials will accrue sufficient patient numbers in this disease setting, and novel study designs, including umbrella, basket, and platform trials, should be considered given the presence of actionable targets. Interestingly, the first reported cohort of the DART trial (ClinicalTrials.gov identifier: NCT02834013)^31^ was the neuroendocrine cohort, with a 44% overall response rate in those with high-grade disease. Ultimately, comprehensive genomic characterization may catalyze the investigation and identification of effective therapies, allowing us to improve oncologic outcomes in this aggressive disease.

## Figures and Tables

**TABLE A1.**
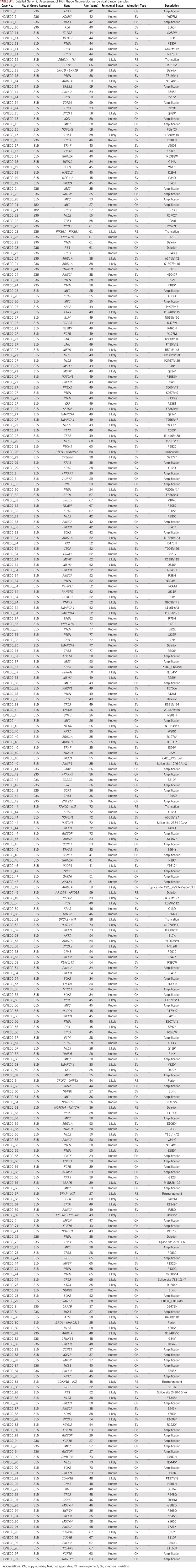
Detailed Genomic Assessment of High Grade Neuroendocrine Cervical Cancer Samples

## Appendix

### Sequencing Methods Used for Comprehensive Genomic Profiling

Sample processing and sequencing were performed in a Clinical Laboratory Improvement Amendments– and College of American Pathologists–accredited laboratory. Briefly, after pathologic review to confirm sufficient tumor nuclei (minimum, 20%) and mitigate pathologic inconsistencies, at least 50 ng of DNA was extracted from 40 microns of tumor samples provided as formalin-fixed, paraffin-embedded tissue blocks. The samples were assayed using adaptor-ligation and hybrid-capture next-generation sequencing (FoundationOne) for all coding exons from 182 (version 1), 287 (version 2), or 315 (version 3) cancer-related genes plus selected introns from 14 (version 1), 19 (version 2), or 28 (version 3) genes frequently rearranged in cancer.

Sequencing of captured libraries was performed using HiSEquation 2500/4000 (Illumina, San Diego, CA) to a mean exon coverage depth of > 500×, and resultant sequences were analyzed using both an algorithmic pipeline and manual curation for base substitutions, small insertions or deletions (indels), copy number alterations (focal amplifications and homozygous deletions), and selected gene fusions, as previously described.^13^ Clinically relevant genomic alterations were defined as alterations targetable by anticancer drugs currently available on the market or in registered clinical trials. Germline variants documented in the dbSNP database (dbSNP142; http://www.ncbi.nlm.nih.gov/SNP/), with ≥ 2 counts in the ExAC database (http://exac.broadinstitute.org/), or recurrent variants of unknown significance that were predicted by an internally developed algorithm to be germline were removed, with the exception of known driver germline events.^12^ Known, confirmed somatic alterations deposited in the Catalog of Somatic Mutations in Cancer (version 62) were highlighted as biologically significant, as were inactivating events in tumor suppressor genes. To maximize mutation-detection accuracy (sensitivity and specificity) in impure clinical specimens, the test was previously optimized and validated to detect base substitutions at a ≥ 5% mutant allele frequency (MAF), indels with a ≥ 10% MAF with ≥ 99% accuracy, and fusions occurring within baited introns/exons with > 99% sensitivity.^12^ Each tumor sample was analyzed alongside an internally validated mixture of 10 heterozygous diploid HapMap control samples, which custom algorithms used to normalize the sequence coverage distribution across baited targets.

## References

[1] Cancer Genome Atlas Research Network: Integrated genomic and molecular characterization of cervical cancer. Nature 543:378-384, 2017 10.1038/nature21386 PMC5354998 28112728

[2] Kandoth C, Schultz N, Cherniack AD, et al: Integrated genomic characterization of endometrial carcinoma. Nature 497:67-73, 2013 [Erratum: Nature 500:242, 2013] 10.1038/nature12113 PMC3704730 23636398

[3] Cancer Genome Atlas Research Network: Integrated genomic analyses of ovarian carcinoma. Nature 474:609-615, 2011 [Erratum: Nature 490:298, 2012] 10.1038/nature10166 PMC3163504 21720365

[4] Gardner GJ Reidy-Lagunes D Gehrig PA Neuroendocrine tumors of the gynecologic tract: A Society of Gynecologic Oncology (SGO) clinical document Gynecol Oncol 122 190 198 2011 21621706 10.1016/j.ygyno.2011.04.011

[5] Satoh T, Takei Y, et al: Gynecologic Cancer InterGroup (GCIG) consensus review for small cell carcinoma of the cervix. Int J Gynecol Cancer 24:S102-S108, 2014 (9 Suppl 3) 10.1097/IGC.0000000000000262 25341572

[6] Fukuoka M Masuda N Furuse K et al A randomized trial in inoperable non-small-cell lung cancer: Vindesine and cisplatin versus mitomycin, vindesine, and cisplatin versus etoposide and cisplatin alternating with vindesine and mitomycin J Clin Oncol 9 606 613 1991 1648597 10.1200/JCO.1991.9.4.606

[7] Kubota K Hida T Ishikura S et al Etoposide and cisplatin versus irinotecan and cisplatin in patients with limited-stage small-cell lung cancer treated with etoposide and cisplatin plus concurrent accelerated hyperfractionated thoracic radiotherapy (JCOG0202): A randomised phase 3 study Lancet Oncol 15 106 113 2014 24309370 10.1016/S1470-2045(13)70511-4

[8] Ishikawa M Kasamatsu T Tsuda H et al Prognostic factors and optimal therapy for stages I-II neuroendocrine carcinomas of the uterine cervix: A multi-center retrospective study Gynecol Oncol 148 139 146 2018 29113721 10.1016/j.ygyno.2017.10.027

[9] Paraghamian SE Longoria TC Eskander RN Metastatic small cell neuroendocrine carcinoma of the cervix treated with the PD-1 inhibitor, nivolumab: A case report Gynecol Oncol Res Pract 4 3 2017 28174665 10.1186/s40661-017-0038-9 PMC5290639

[10] Sharabi A Kim SS Kato S et al Exceptional response to nivolumab and stereotactic body radiation therapy (SBRT) in neuroendocrine cervical carcinoma with high tumor mutational burden: Management considerations from the Center for Personalized Cancer Therapy at UC San Diego Moores Cancer Center Oncologist 22 631 637 2017 28550027 10.1634/theoncologist.2016-0517 PMC5469598

[11] Foundation Medicine: FoundationOne CDx. http://foundationone.com/docs/FoundationOne_tech-info-and-overview.pdf

[12] Frampton GM Fichtenholtz A Otto GA et al Development and validation of a clinical cancer genomic profiling test based on massively parallel DNA sequencing Nat Biotechnol 31 1023 1031 2013 24142049 10.1038/nbt.2696 PMC5710001

[13] Ross JS Fakih M Ali SM et al Targeting HER2 in colorectal cancer: The landscape of amplification and short variant mutations in ERBB2 and ERBB3 Cancer 124 1358 1373 2018 29338072 10.1002/cncr.31125 PMC5900732

[14] Chalmers ZR Connelly CF Fabrizio D et al Analysis of 100,000 human cancer genomes reveals the landscape of tumor mutational burden Genome Med 9 34 2017 28420421 10.1186/s13073-017-0424-2 PMC5395719

[15] Johnson DB Frampton GM Rioth MJ et al Targeted next generation sequencing identifies markers of response to PD-1 blockade Cancer Immunol Res 4 959 967 2016 27671167 10.1158/2326-6066.CIR-16-0143 PMC5134329

[16] Lechner M Frampton GM Fenton T et al Targeted next-generation sequencing of head and neck squamous cell carcinoma identifies novel genetic alterations in HPV+ and HPV- tumors Genome Med 5 49 2013 23718828 10.1186/gm453 PMC4064312

[17] Walsh CS Two decades beyond BRCA1/2: Homologous recombination, hereditary cancer risk and a target for ovarian cancer therapy Gynecol Oncol 137 343 350 2015 25725131 10.1016/j.ygyno.2015.02.017

[18] Chae YK Pan AP Davis AA et al Path toward precision oncology: Review of targeted therapy studies and tools to aid in defining “actionability” of a molecular lesion and patient management support Mol Cancer Ther 16 2645 2655 2017 29203694 10.1158/1535-7163.MCT-17-0597

[19] Carey JPW Keyomarsi K Leveraging MYC as a therapeutic treatment option for TNBC Oncoscience 5 137 139 2018 30035166 10.18632/oncoscience.424 PMC6049306

[20] Tempfer CB Tischoff I Dogan A et al Neuroendocrine carcinoma of the cervix: A systematic review of the literature BMC Cancer 18 530 2018 29728073 10.1186/s12885-018-4447-x PMC5935948

[21] George J Lim JS Jang SJ et al Comprehensive genomic profiles of small cell lung cancer Nature 524 47 53 2015 26168399 10.1038/nature14664 PMC4861069

[22] Patel M Kato SM Kurzrock R Molecular tumor boards: Realizing precision oncology therapy Clin Pharmacol Ther 103 206 209 2018 29134641 10.1002/cpt.920 PMC5760337

[23] Kato S, Ross JS, Gay L, et al:Analysis of MDM2 amplification: Next-generation sequencing of patients with diverse malignancies. JCO Precis Oncol 10.1200/PO.17.00235 10.1200/PO.17.00235 PMC6106866 30148248

[24] Kato S Kurasaki K Ikeda S et al Rare Tumor Clinic: The University of California San Diego Moores Cancer Center experience with a precision therapy approach Oncologist 23 171 178 2018 29038235 10.1634/theoncologist.2017-0199 PMC5813742

[25] Hainsworth JD Meric-Bernstam F Swanton C et al Targeted therapy for advanced solid tumors on the basis of molecularp: Results from MyPathway, an open-label, phase IIa multiple basket study J Clin Oncol 36 536 542 2018 29320312 10.1200/JCO.2017.75.3780

[26] Janku F Hong DS Fu S et al Assessing PIK3CA and PTEN in early-phase trials with PI3K/AKT/mTOR inhibitors Cell Rep 6 377 387 2014 24440717 10.1016/j.celrep.2013.12.035 PMC4409143

[27] Tsimberidou AM, Hong DS, Ye Y, et al: Initiative for Molecular Profiling and Advanced Cancer Therapy (IMPACT): An MD Anderson precision medicine study. JCO Precis Oncol 10.1200/PO.17.00002 10.1200/PO.17.00002 PMC5659750 29082359

[28] Goodman AM Kato S Bazhenova L et al Tumor mutational burden as an independent predictor of response to immunotherapy in diverse cancers Mol Cancer Ther 16 2598 2608 2017 28835386 10.1158/1535-7163.MCT-17-0386 PMC5670009

[29] Alldredge JK Eskander RN EZH2 inhibition in *ARID1A* mutated clear cell and endometrioid ovarian and endometrioid endometrial cancers Gynecol Oncol Res Pract 4 17 2017 29093822 10.1186/s40661-017-0052-y PMC5663065

[30] Alejo M Alemany L Clavero O et al Contribution of human papillomavirus in neuroendocrine tumors from a series of 10,575 invasive cervical cancer cases Papillomavirus Res 5 134 142 2018 29555602 10.1016/j.pvr.2018.03.005 PMC5909066

[31] 10.1158/1538-7445.AM2019-CT039

